# Study on the risk factors for colorectal polyp recurrence: a cross-sectional retrospective cohort study

**DOI:** 10.3389/fmed.2025.1553194

**Published:** 2025-06-18

**Authors:** Fang Li, Mengge Lu, Bo Xu, Zheng Xiang, Yuan Zhang, Boran Cao, Xuewei Li, Yanan Wu, Rongrong Zheng, Qin Cai, Jun Shen, Pengfei Xin, Lianbo Xiao, Yanqin Bian

**Affiliations:** ^1^Department of Gastroenterology, Guanghua Hospital Affiliated to Shanghai University of Traditional Chinese Medicine, Shanghai, China; ^2^Shanghai Guanghua Hospital of Integrative Medicine, Shanghai, China; ^3^The Research Institute for Joint Diseases, Shanghai Academy of Traditional Chinese Medicine, Shanghai, China

**Keywords:** risk factors, recurrence, a cross-sectional retrospective cohort study, risk prediction model, colorectal polyps

## Abstract

**Objective:**

To investigate the factors associated with the recurrence of colorectal polyps.

**Methods:**

Data on polyp recurrence and related factors, including gender, age, BMI, family history, smoking history, alcohol consumption history, gallbladder disease history, food allergy, polyp size, number, and pathological classification, *Helicobacter pylori* (Hp) infection, parathyroid hormone, gastrin, and blood lipid levels, were collected as exposure factors. Polyp recurrence was used as the outcome measure. Logistic regression analysis was used to evaluate risk and protective factors for colorectal polyp recurrence. The diagnostic performance of the identified risk factor model was assessed using ROC curve analysis.

**Results:**

Among the 318 patients, 170 experienced polyp recurrence, while 148 did not. Logistic regression analysis revealed that gender (OR = 1.927, 95% CI = 1.134–3.276, *P* = 0.015), age_60–80__years_ (OR = 3.228, 95% CI = 1.846–5.647, *P* < 0.001), history of gallbladder disease (OR = 2.011, 95% CI = 1.147–3.523, *P* = 0.015), food allergy (OR = 2.246, 95% CI = 1.211–4.545, *P* = 0.012), pathological classification (OR = 5.023, 95% CI = 2.932–8.606, *P* < 0.001), and Hp infection (OR = 1.970, 95% CI = 1.171–3.312, *P* = 0.011) were positively associated with polyp recurrence. Conversely, polyp size (OR = 0.324, 95% CI = 0.127–0.827, *P* = 0.018) was negatively associated with recurrence. Logit(p) = −2.459 + 0.656 × Gender + 1.172 × Age 61–80 years + 0.698 × Gallbladder Disease + 0.853 × Food Allergy−1.127 × Polyp Size + 1.164 × Pathological Classification + 0.678 × Hp Infection. The risk prediction model can be used to predict post-surgical recurrence of colorectal polyps with a sensitivity of 0.88 and specificity of 0.56. The cutoff value for this odds prediction model is 0.44.

**Conclusion:**

Elderly (61–80 years old) male patients with adenomatous colorectal cancer and the history of *Helicobacter pylori* (Hp) infection, gallbladder disease and food allergy have higher odds to experience recurrence after surgical resection. On the contrary, those patients with a larger polyp size (≥2 cm) are less odds to experience recurrence. Patients with a risk prediction model value greater than or equal to 0.44 have increased odds to experience postoperative recurrence.

## 1 Introduction

Colorectal polyps are growths on the colonic mucosa that can cause symptoms such as abdominal pain, diarrhea, constipation, rectal bleeding, and mucus in the stool. In some cases, however, there may be no obvious symptoms (1). The most common type of colorectal polyp is the adenomatous polyp, which is also the most significant precancerous lesion for colorectal cancer (CRC). Research shows that 85%–90% of colorectal cancers develop from adenomas, and the polyp-adenoma-cancer sequence is now widely recognized as the primary pathway for colorectal cancer progression (2, 3). Colorectal cancer ranks as the third most common cancer worldwide and is the fourth leading cause of cancer-related deaths, posing a significant threat to human health (4). In China, the incidence and mortality of colorectal cancer are rising annually, with a growing trend among younger populations. Studies indicate that even after polyp removal, patients still face a relatively high risk of recurrence or malignant transformation (5), underscoring the importance of early prevention and treatment to reduce the risk of colorectal cancer. Even after endoscopic polyp removal, the recurrence rate remains as high as 30%–50% (6–8). The *Delphi Initiative for the Early-Onset Colorectal Cancer (DIRECt) International Management Guidelines* (9) highlights relevant risk factors, including hereditary CRC syndromes, long-term inflammatory bowel disease (10, 11), ethnicity (12), sugar intake (13, 14), obesity (15, 16), gender, hyperlipidemia, smoking, alcohol consumption (17), dietary habits and lifestyle (18), among others (19, 20). However, the evidence for these associations remains controversial. Consequently, many experts recommend combining risk factors into a comprehensive risk score, but no standardized framework for clinical application has yet been established.

This study utilized a cross-sectional retrospective cohort design to investigate patients who underwent colorectal polyp resection, with a follow-up period of 1 year. Exposure factors included patient characteristics at the time of surgery, such as gender, age, BMI, family history, smoking history, alcohol consumption history, history of gallbladder disease, food allergy, polyp size, number, pathological classification, *Helicobacter pylori* infection, parathyroid hormone, gastrin, and blood lipid levels. The outcome measure was defined as polyp recurrence within one year postoperatively. This study aims to explore the risk factors for colorectal polyp recurrence in depth, providing new perspectives for the prevention and treatment of patients with colorectal polyps.

## 2 Materials and methods

### 2.1 Patients

**FIGURE 1:**
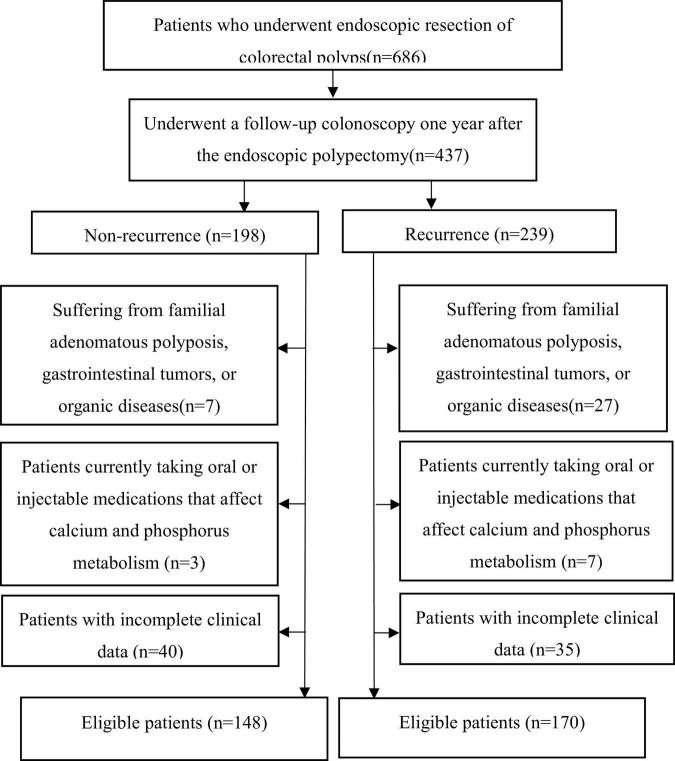
The flowchart of the study.

We retrospectively screened 686 patients who underwent endoscopic colorectal polyp resection between 1 January 2022, and 1 September 2024. Among these, 437 patients underwent follow-up colonoscopy one year postoperatively. According to the with recurrence or not, all subjects were divided into two groups, recurrence group (*n* = 239), Non-recurrence group (*n* = 198). After applying exclusion criteria, 318 patients (148 non-recurrence and 170 recurrence) met the inclusion criteria and completed the study. The study flowchart is shown in Figure 1. All patients were diagnosed with colorectal polyps and underwent endoscopic resection of colorectal polyps. This study was approved by the Ethics Committee of Guanghua Hospital Affiliated to Shanghai University of Traditional Chinese Medicine (2021-K-37). The inclusion requirements were as follows: (1) Individuals who satisfied the diagnostic criteria for colorectal polyps in *2024 ESGE Guideline* (21) and the *2021 Colorectal Cancer Screening Guidelines* (22) and had undergone endoscopic resection; (2) No restrictions on gender or age; (3) Patients with clear consciousness who were able to cooperate in the collection of clinical data and related laboratory tests; (4) Patients who provided written informed consent to participate in the study. The exclusion criteria were as follows: (1) Patients with other significant intestinal diseases, familial adenomatous polyposis, or confirmed malignancies; (2) those with pathological findings of high-grade intraepithelial neoplasia, suspected malignancies, or confirmed malignant changes; (3) those with severe dysfunction of the heart, lungs, liver, or kidneys; compromised immune function; or malignant tumors; (4) those who had previously undergone gastrectomy; (5) those currently participating in other clinical trials; (6) female patients planning to conceive, currently pregnant, breastfeeding, or those with mental disorders or unable to complete the study. As shown in Figure 1, the process of including or excluding patients is summarized.

### 2.2 Exposure factors

The exposure factors analyzed in this study include: (1) Patient Information and Medical History: gender, age, BMI, family history, smoking history, alcohol consumption history, and history of gallbladder disease. (2) Laboratory Test Results: food allergen analysis, polyp size, number and pathological classification, 13C urea breath test, parathyroid hormone levels, gastrin levels, and blood lipid profiles. These exposure factors were included as independent variables to explore their association with polyp recurrence.

### 2.3 Outcome measures

After one year of follow-up, patients underwent a repeat colonoscopy to evaluate their postoperative recovery status. Following the recommendations from the *Asia-Pacific Consensus on Colorectal Cancer Screening* (23), recurrence was determined based on polyp presence, recording their number, size, and pathological nature.

### 2.4 Establishment of the predictive model

A logistic regression analysis was conducted to identify factors associated with polyp recurrence. A total of 14 factors, including: gender, age, BMI, smoking history, drinking history, history of gallbladder disease (based on the abdominal ultrasound report), *Helicobacter pylori* (Hp) status, polyp size, polyp number, pathological classification, abnormal blood lipid levels, gastrin levels, parathyroid hormone levels and food allergy were considered as independent variables (X), and whether polyp recurrence occurred as dependent variable (Y). The backward LR method with the entry probability at 0.10 and the removal probability at 0.15 was used to construct a multivariable logistic regression model, with details of risk factors and their coding presented in Supplementary Table 1. Then the multivariable logistic regression model was used to estimate the probability of postoperative polyp recurrence by ROC curve (AUC) analysis.

### 2.5 Statistical analysis

Statistical analysis was conducted using SPSS 26.0 software, following a stepwise approach. Continuous variables were expressed as mean±standard deviation (x±s). Categorical variables were expressed as numbers and percentages and analyzed using the χ^2^ test. All tests were two-tailed unless otherwise specified, with *P* < 0.05 considered statistically significant. For normally distributed variables with homogeneity of variance, independent samples *t*-tests were used. For normally distributed variables with heterogeneity of variance, approximate *t*’ tests were applied. For non-normally distributed variables, the Wilcoxon rank-sum test was used.

A logistic regression analysis was conducted to identify factors associated with polyp recurrence. The Backward LR method with the entry probability at 0.10 and the removal probability at 0.15 was used to construct a multivariable logistic regression model. *p*-value of LR test < 0.0001 indicates that the model is statistically significant overall.

## 3 Results

### 3.1 Patient characteristics

The study included a total of 318 patients. Among them, 148 cases were categorized as non-recurrence, and 170 cases as recurrence. Detailed patient information is presented in Table 1. The risk factors and corresponding variable assignments are listed in Supplementary Table 1. Except for age and pathological classification, there were no statistically significant differences in the baseline comparisons of other exposure factors between the recurrence and non-recurrence groups.

**TABLE 1:** Base characteristics of patients in the non-recurrence set and recurrence set.

Feature[*n* (%)]	Category	Non-recurrence (*n* = 148)	Recurrence (*n* = 170)	*P*
Sex	Female	73 (49.3%)	78 (45.9%)	0.617
	Male	75 (50.7%)	92 (54.1%)	
Age	20–40 years	15 (10.1%)	7 (4.1%)	0.003
	41–60 years	42 (28.4%)	30 (17.6%)	
	61–80 years	82 (55.4%)	127 (74.7%)	
	> 80 years	9 (6.1%)	6 (3.5%)	
BMI (kg/m^2^)	< 18.5	6 (4.1%)	3 (1.8%)	0.234
	18.5–23.9	96 (64.9%)	98 (57.6%)	
	24–26.9	38 (25.7%)	55 (32.4%)	
	> 27	8 (5.4%)	14 (8.2%)	
Family history of polyp	No	60 (40.5%)	67 (39.4%)	0.928
	Yes	88 (59.5%)	103 (60.6%)	
History of gallbladder diseases	No	102 (68.9%)	108 (63.5%)	0.372
	Yes	46 (31.1%)	62 (36.5%)	
History of smoking	No	110 (74.3%)	120 (70.6%)	0.537
	Yes	38 (25.7%)	50 (29.4%)	
History of drinking	No	111 (75.0%)	131 (77.1%)	0.766
	Yes	37 (25.0%)	39 (22.9%)	
Food allergy	No	107 (72.3%)	106 (62.4%)	0.078
	Yes	41 (27.7%)	64 (37.6%)	
Number of polyps	< 3	65 (43.9%)	64 (37.6%)	0.307
	≥3	83 (56.1%)	106 (62.4%)	
Size of polyps	< 2 cm	126 (85.1%)	154 (90.6%)	0.186
	≥ 2 cm	22 (14.9%)	16 (9.4%)	
Pathological classification	Non-adenomatous	94 (63.5%)	58 (34.1%)	< 0.001
	Adenomatous	54 (36.5%)	112 (65.9%)	
*H. pylori* infection	Negative	91 (61.5%)	89 (52.4%)	0.127
	Positive	57 (38.5%)	81 (47.6%)	
PTH levels	Normal	88 (59.5%)	103 (60.6%)	0.928
	Abnormal	60 (40.5%)	67 (39.4%)	
G17 levels	Normal	13 (8.8%)	21 (12.4%)	0.398
	Abnormal	135 (91.2%)	149 (87.6%)	
Blood lipid levels	Normal	53 (35.8%)	53 (31.2%)	0.450
	Abnormal	95 (64.2%)	117 (68.8%)	

SD, standard deviation; BMI, body mass index; *H. pylori*, *Helicobacter pylori*; PTH, parathyroid hormone; G17, gastrin-17; OR, odds ratio; CI, confidence interval.

### 3.2 Elderly male patients with adenomatous colorectal cancer are higher odds to experience recurrence after surgical resection

**FIGURE 2:**
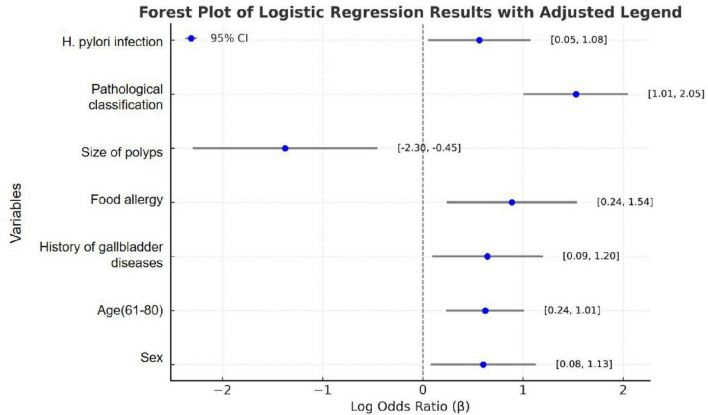
Forest plot of logistic regression.

To identify significant risk factors for recurrence following colorectal cancer resection, we employed the Logistic stepwise regression method (Backward LR). The analysis used the following parameters: Constant Term, −3.2043; Pseudo R^2^, 0.1399; Log-Likelihood, −188.93. Logistic regression analysis identified several significant factors associated with colorectal polyp recurrence. As shown in Table 2 and Figure 2, male patients (OR = 1.927, 95% CI = 1.134–3.276, *P* = 0.015) had higher odds to experience recurrence compared to female patients, corresponding to an 92.7% increased odds. The odds of recurrence in patients aged 61–80 years is 3.228 times higher than that in the 20–40-year-old group (OR = 3.228, 95% CI = 1.846–5.647, *P* < 0.001). Patients with a history of gallbladder disease have 2.011 times higher odds of recurrence compared to those without such a history, indicating a 101.1% increased odds (OR = 2.011, 95% CI = 1.147–3.523, *P* = 0.015). Patients with food allergies had a 2.246-fold higher odds of recurrence compared to those without allergies, equating to a 124.6% increased odds (OR = 2.246, 95% CI = 1.211–4.545, *P* = 0.012). Patients with adenomatous polyps had 5.023 times higher odds to experience recurrence compared to those with non-adenomatous polyps, representing a 402.3% increased odds (OR = 5.023, 95% CI = 2.932–8.606, *P* < 0.001). Patients with *Helicobacter pylori* infection had 1.970 times higher odds to experience recurrence compared to uninfected patients, equating to a 97.0% increased risk (OR = 1.970, 95% CI = 1.171–3.312, *P* = 0.011). Conversely, patients with larger polyps (≥2 cm) had 0.324 times higher adds to experience recurrence compared to those with smaller polyps (<2 cm), indicating a 67.6% reduction in recurrence risk (OR = 0.324, 95% CI = 0.127–0.827, *P* = 0.018). The results indicate that elderly male patients are higher odds to experience recurrence after surgery. A history of food allergies significantly increases the odds of recurrence, while the presence of polyps larger than 2 cm at the time of surgical resection serves as a protective factor for patient prognosis.

**TABLE 2:** Univariable and multivariable logistic regression analyses for predicting factors associated with polyp recurrence in the polyp patients.

	Univariable				Multivariable			
	β	*P*-value	OR	95% CI	β	*P*-value	OR	95% CI
Sex (female/male)	1.067	0.004	2.908	1.419–5.957	0.656	0.015	1.927	1.134–3.276
Age (20–40 years)	–	–	–	–				
Age (41–60 years)	0.868	0.144	2.383	0.744–7.629				
Age (61–80 years)	2.156	0.000	8.641	2.695–27.703	1.172	< 0.001	3.228	1.846–5.647
Age (> 80 years)	0.833	0.303	2.299	0.472–11.204				
BMI (< 18.5 kg/m^2^)	–	–	–	–				
BMI (18.5–23.9 kg/m^2^)	0.934	0.305	2.544	0.427–15.173				
BMI (24–26.9 kg/m^2^)	1.245	0.179	3.472	0.565–21.345				
BMI (> 27 kg/m^2^)	0.570	0.582	1.768	0.233–13.426				
Family history (yes/no)	0.327	0.240	1.387	0.804–2.394				
History of gallbladder disease (yes/no)	0.729	0.020	2.073	1.120–3.839	0.698	0.015	2.011	1.147–3.523
History of smoking (yes/no)	−0.598	0.103	0.550	0.268–1.130				
History of drinking (yes/no)	−0.234	0.496	0.791	0.403–1.552				
Food allergy (yes/no)	0.900	0.014	2.461	1.200–5.048	0.853	0.012	2.346	1.211–4.545
Number of polyps (< 3/ ≥ 3)	−0.141	0.637	0.868	0.483–1.562				
Size of polyps (< 2 cm/ ≥ 2 cm)	−1.063	0.042	0.346	0.124–0.963	−1.127	0.018	0.324	0.127–0.827
Pathological classification (Non-ade/ade)	1.758	< 0.001	5.801	3.252–10.350	1.614	< 0.001	5.023	2.932–8.606
*H. pylori* infection (negative/positive)	0.714	0.012	2.043	1.172–3.561	0.678	0.011	1.970	1.171–3.312
PTH (normal/abnormal)	−0.047	0.838	0.954	0.612–1.501				
G17 (normal/abnormal)	−0.056	0.904	0.945	0.379–2.359				
Blood lipids (normal/abnormal)	0.033	0.910	1.034	0.583–1.834				

BMI, body mass index; *H. pylori*, *Helicobacter pylori*; PTH, parathyroid hormone; G17, gastrin-17; OR, odds ratio; CI, confidence interval.

In this study,

### 3.3 A predictive model for assessing postoperative recurrence risk of colorectal polyps

To further explore risk factors for predicting postoperative recurrence of colorectal polyps, we constructed a risk prediction model based on the identified factors and evaluated its diagnostic performance. The predictive logistic regression model for postoperative recurrence of colorectal polyps is as follows: Logit(p) = −2.459 + 0.656 × Gender + 1.172 × Age 61–80 years + 0.698 × Gallbladder Disease + 0.853 × Food Allergy−1.127 × Polyp Size + 1.164 × Pathological Classification + 0.678 × Hp Infection.

Gender: Male = 1, Female = 0;

Age: Patient’s age (20–40 years = 0, 0, 0, 0; 41–60 years = 0, 1, 0, 0; 61–80 years = 0, 0, 1, 0; > 80 = 0, 0, 0, 1);

Gallbladder Disease: History of gallbladder disease (Yes = 1, No = 0);

Food Allergies: Presence of food allergies (Yes = 1, No = 0);

Polyp Size: Size of the polyp (> 2 cm = 1, < 2 cm = 0)

Pathological Classification: Adenomatous = 1, Non-adenomatous = 0;

*H. pylori* Infection: Presence of *H. pylori* infection (Yes = 1, No = 0).

**FIGURE 3:**
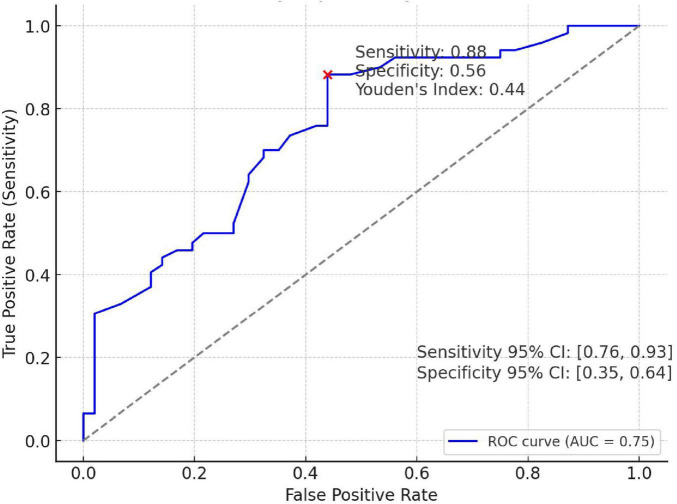
ROC curve for colorectal polyp recurrence.

This model can be used to estimate the probability of postoperative polyp recurrence based on individual patient characteristics. As shown in Figure 3, area under the ROC curve (AUC) of the model was 0.75, with the sensitivity was 0.88, the specificity was 0.56, and Youden’s index = 0.44. The results indicate that colorectal polyp patients with predicted model values greater than or equal to 0.44 are more odds to experience postoperative recurrence.

## 4 Discussion

Early prevention and treatment of colorectal polyps are critical in preventing colorectal cancer. Currently, colorectal cancer is primarily prevented through the endoscopic removal of polyps (24). However, even after endoscopic polypectomy or treatment, preventive measures are necessary to reduce the risk of recurrence or malignant transformation (22). The clinical field is actively seeking strategies tailored to individuals with varying risk levels. Preventing the development and progression of polyps by targeting risk factors is one of the urgent challenges in current clinical research.

Our study identified male gender as a risk factor for colorectal polyp recurrence. Males were found to have a significantly higher recurrence odds compared to females. This elevated odds may be associated with lifestyle choices, hormonal levels, and metabolic factors. Research suggests that men often consume higher levels of fat and lower amounts of dietary fiber, which may increase intestinal inflammation and polyp formation. Androgens may further influence gut microbiota and inflammation, promoting polyp recurrence (25). A positive correlation was observed between older age and polyp recurrence. Elderly patients (> 60 years old) require enhanced postoperative monitoring. Age-related cumulative genetic and epigenetic changes, such as mutations in the APC, KRAS, and TP53 genes, contribute to the adenoma-carcinoma sequence (26, 27). Additionally, immunosenescence and gut microenvironment alterations, such as dysbiosis, further exacerbate the recurrence risk in older patients (28).

Gallbladder diseases, especially chronic cholecystitis, are associated with chronic inflammation in the biliary system. This inflammatory response may alter bile composition, impact intestinal bile acid metabolism, and induce local intestinal inflammation and mucosal hyperplasia, thereby promoting polyp recurrence and malignant transformation (29, 30). Our founding indicate that among the 108 patients with a history of cholecystitis, the recurrence rate was significantly higher compared to those without prior cholecystitis. Patients with food allergy had a higher odds of recurrence compared to non-allergic individuals. Food allergy result in overactivation of the immune system, particularly Th2-mediated immune responses, leading to chronic inflammation and impaired intestinal mucosal barrier function. Cytokines such as IL-4 and IL-13 released during immune activation may promote mucosal hyperplasia, accelerating polyp formation and recurrence (31).

Pathological classification emerged as the strongest predictor of polyp recurrence. Adenomatous polyps were significantly more odds to recur than non-adenomatous polyps. Adenomatous polyps exhibit higher proliferation and dysplasia, and their progression is closely linked to activation of the Wnt/β-catenin signaling pathway. Additionally, high-grade adenomas are prone to abnormal cell proliferation and genomic instability, increasing the odds of recurrence or malignant transformation (32). *H. pylori* infection was significantly associated with polyp recurrence, consistent with findings from other studies (33). *H. pylori* induces chronic gastritis, disrupts the gastrointestinal mucosal barrier, and leads to abnormal gastric acid secretion, altering the gut microenvironment. Furthermore, *H. pylori* infection is linked to the release of gastrointestinal inflammatory mediators such as TNF-α and IL-8, which may promote polyp formation and recurrence (34, 35). Interestingly, larger polyps (≥2 cm) were associated with a lower odds of recurrence (36). This may be due to the slower growth cycle of larger polyps. Given the 1-year follow-up period in this study, smaller polyps may have a higher odds of recurrence within this timeframe. These findings provide important evidence for risk stratification and targeted interventions aimed at reducing recurrence rates and improving patient outcomes. Strategies such as eradication therapy for *H. pylori* infection and dietary adjustments for food allergy patients may help mitigate recurrence risks. In the present study, no significant association was found between parathyroid hormone (PTH), gastrin, or serum lipid levels and the recurrence of colorectal polyps, despite existing literature reporting correlations between these factors and colorectal polyp recurrence (37–39). This discrepancy may stem from our methodological approach of dichotomizing these parameters (normal vs. abnormal) rather than incorporating continuous numerical values, which consequently excluded these biomarkers from the final analytical model.

Despite the rigorous statistical analysis in this study, certain limitations remain. First, as a retrospective study, there is a potential for selection bias. Second, unmeasured confounding factors, such as genetic predisposition, may have influenced the results. Future research should focus on prospective validation of these findings in larger cohorts. Integrating genetic and molecular biomarkers with clinical factors may enhance the accuracy of recurrence prediction models.

## 5 Conclusion

Elderly (60–80 years) male patients with adenomatous colorectal cancer and the history of *Helicobacter pylori* (Hp) infection, gallbladder disease and food allergy have higher odds to experience recurrence after surgical resection. On the contrary, those patients with a larger polyp size (≥2 cm) are less odds to experience recurrence. When the colorectal polyp patients with predicted model value is greater than or equal to 0.44, they have higher odds to experience postoperative recurrence and need to be paid more attentions.

The findings of this study provide a valuable predictive tool for clinically estimating the probability of recurrence in postoperative colon cancer patients. The results will guide gastroenterologists in evaluating the long-term outcomes of patients after colon cancer surgery and better inform postoperative rehabilitation strategies, demonstrating significant application value in clinical practice.

## Data Availability

The raw data supporting the conclusions of this article will be made available by the authors, without undue reservation.
