# Supplementary material for: Study on the risk factors for colorectal polyp recurrence: a cross-sectional retrospective cohort study
Source: Front Med (Lausanne). 2025 Jun 18;12:1553194. doi: 10.3389/fmed.2025.1553194 (PMC12213858; doi:10.3389/fmed.2025.1553194)
Supplement: Supplementary file 1 [file Table_1.docx]

Supp. Table 1 The risk factors and corresponding variable assignments

| **Factor** | **Variable Name** | **Assignment Description** |
| --- | --- | --- |
| **Group** | Y | 0 = Non-recurrence, 1 = Recurrence |
| **Sex** | X_1_ | 0 = Female, 1 = Male |
| **Age** | X_2_ | X_2-1_:0=20-40years,0=41-60years, 0=61-80years, 0= >80 years  X_2-2_:0=20-40years,1=41-60years, 0=61-80years, 0= >80 years  X_2-3_:0=20-40years,0=41-60years, 1=61-80years, 0= >80 years  X_2-4_:0=20-40years,0=41-60years, 0=61-80years, 1= >80 years |
| **BMI classification**  **(kg/m²)** | X_3_ | X3-1:0=<18.5,0=18.5-23.9,0=24-26.9, 0= Obesity >27  X3-2:0=<18.5,1=18.5-23.9,0=24-26.9, 0= Obesity >27  X3-3:0=<18.5,0=18.5-23.9,1=24-26.9, 0= Obesity >27  X3-4:0=<18.5,0=18.5-23.9,0=24-26.9, 1= Obesity >27 |
| **Family history** | X_4_ | 0 = No, 1 = Yes |
| **History of gallbladder disease** | X_5_ | 0 = No, 1 = Yes |
| **Smoking history** | X_6_ | 0 = No, 1 = Yes |
| **Drinking history** | X_7_ | 0 = No, 1 = Yes |
| **Food allergy** | X_8_ | 0 = No, 1 = Yes |
| **Number of polyps** | X_9_ | 0 = <3, 1 = ≥3 |
| **Polyp size** | X_10_ | 0 = <2cm, 1 = ≥2cm |
| **Pathological classification** | X_11_ | 0 = Non-adenomatous, 1 = Adenomatous |
| **H. Pylori infection** | X_12_ | 0 = Negative, 1 = Positive |
| **PTH** | X_13_ | 0 = Normal, 1 = Abnormal |
| **Gastrin 17 (G17)** | X_14_ | 0 = Normal, 1 = Abnormal |
| **Blood lipids** | X_15_ | 0 = Normal, 1 = Abnormal |
